# Characteristics of Older Adults Who Were Early Adopters of Medical Cannabis in the Florida Medical Marijuana Use Registry

**DOI:** 10.3390/jcm9041166

**Published:** 2020-04-18

**Authors:** Joshua D. Brown, Brianna Costales, Sascha van Boemmel-Wegmann, Amie J. Goodin, Richard Segal, Almut G. Winterstein

**Affiliations:** 1 Center for Drug Evaluation & Safety, Department of Pharmaceutical Outcomes & Policy, University of Florida College of Pharmacy, Gainesville, FL 32610, USA; b.costales@ufl.edu (B.C.); wegmann@ufl.edu (S.v.B.-W.); amie.goodin@ufl.edu (A.J.G.); segal@ufl.edu (R.S.); almut@ufl.edu (A.G.W.); 2 Consortium for Medical Marijuana Clinical Outcomes Research, Center for Drug Evaluation & Safety, Department of Pharmaceutical Outcomes & Policy, University of Florida College of Pharmacy, Gainesville, FL 32610, USA; 3 Department of Epidemiology, University of Florida College of Colleges of Public Health and Health Professions and Medicine, Gainesville, FL 32610, USA

**Keywords:** medical marijuana, cannabis, cannabidiol, CBD, THC, tetrahydrocannabinol, older adults, safety, effectiveness

## Abstract

Use of medical marijuana is increasing in the United States and older adults are the fastest growing user group. There is little information about the characteristics and outcomes related to medical marijuana use. This study is a descriptive analysis of older adults (aged ≥50 years old) who were early adopters of a medical marijuana program in the U.S. state of Florida. Per state legislation, initial and follow-up treatment plans were submitted to the University of Florida College of Pharmacy. Data collection included demographics, clinical history, medical conditions, substance use history, prescription history, and health status. Follow-up treatment plans noted changes in the chief complaint and actions taken since the initial visit. Of the state’s 7548 registered users between August 2016 and July 2017, *N* = 4447 (58.9%) were older adults. Patients utilized cannabidiol (CBD)-only preparations (45%), preparations that had both tetrahydrocannabinol (THC) and CBD (33.3%) or were recorded to use both CBD-only and THC + CBD products (21.7%). The chief complaints indicating medical cannabis treatment were musculoskeletal disorders and spasms (48.4%) and chronic pain (45.4%). Among other prescription medications, patients utilized antidepressants (23.8%), anxiolytics and benzodiazepines (23.5%), opioids (28.6%), and cardiovascular agents (27.9%). Among all drug classes with potential sedating effects, 44.8% of the cohort were exposed to at least one. Patients with follow-up visits (27.5%) exhibited marked improvement as assessed by the authorizing physicians. However, the patient registry lacked detailed records and linkable information to other data resources to achieve complete follow up in order to assess safety or efficacy. Future improvements to registries are needed to more adequately capture patient information to fill knowledge gaps related to the safety and effectiveness of medical marijuana, particularly in the older adult population.

## 1. Introduction

Cannabis use is increasing among medically complex individuals. The vast majority of cannabis use is recreational; however, there is an increasing number of adults who use cannabis and cannabis-derived substances for medical and complementary health purposes. Increased use corresponds with expanding access through state medical cannabis programs, broad consumer marketing and use of cannabidiol (CBD) products. There is a continued increase in public support of legalization at the individual state level, whereas cannabis remains illegal (i.e., Schedule 1) at the national level [1,2,3]. State programs range from what is deemed a “comprehensive” program that allows both CBD and tetrahydrocannabinol (THC) use (*N* = 33 states), programs that restrict the amount of THC allowed and promote CBD-only products (*N* = 13 states), and four states (ID, SD, NE, KS) which have no program in place [1]. Currently, 11 states have also legalized recreational cannabis for use by adults [1]. Florida was the 22nd state in the U.S. to legalize access to medical marijuana—the third largest state with one of the largest and fastest growing populations of older adults.

In 2018, state-based medical cannabis programs were estimated to include over 2.1 million legal medical cannabis patients. Enrollment in these programs varies by state, with a range of 1 to >38 patients per 1000 residents [4]. Medical cannabis users represent approximately 10% of adult cannabis users [5]. Practically all state programs have specified conditions for which medical cannabis can be used. These conditions include epilepsy/seizures, chronic pain, nausea/vomiting, muscle spasms, inflammatory conditions, Alzheimer’s disease, Parkinson’s disease, and cancer [1]. These conditions are highly prevalent among older adults who are likely to have complex medical profiles and pharmacotherapeutic regimens [6,7,8,9,10]. A recent national survey reported increased odds of about 50% for past year marijuana use among patients with history of stroke, heart disease, asthma, chronic pulmonary disease, diabetes, arthritis, renal disease, cancer, and depression among medical cannabis users [11].

The Baby Boomer generation (~55–75 years old), which represents the fastest growing segment of the population in terms of substance use and abuse in general, are more likely to be comfortable with cannabis use compared to their parents’ generation due to social or personal exposure earlier in life [12,13]. Data from the period 2006–2013 suggest a 250% increase in cannabis use among those 65+ and nearly a 60% increase among those 50–64 years old—numbers that are likely to have increased as more states legalize medical and recreational cannabis programs [9]. Prevalence estimates of cannabis use in the past year for these age groups are approximately 3% and 9%, respectively [14]. Among older adults, 75% consider cannabis use to have no or only slight health risks if used once or twice a week [14]. Thus, it appears that older adults regard cannabis as generally safe and are rapidly adopting cannabis into their health and medical regimens.

This study described the characteristics of older adult patients, aged ≥50 years old, who were licensed to use medical marijuana during the early implementation period of the Florida medical marijuana program between 2016 and 2017 and followed these individuals from treatment initiation to the point of a follow-up encounter.

## 2. Methods

This was a retrospective analysis of initial and follow-up treatment plan forms electronically submitted by providers to the University of Florida College of Pharmacy (UF-COP) between 01 August 2016 and 31 July 2017. The forms were created to meet the Compassionate Medical Cannabis Act (CCA) statutory requirements by a team of outcomes researchers, health policy experts and physicians and pharmacists with expertise in psychiatry, neurology, and pain medicine. The authorizing physician completed the initial and follow-up treatment plans and submitted the forms electronically via a secured portal maintained by the UF-COP. The forms received covered visit dates during the period in which CBD-only cannabis was available associated with the 2014 CCA legislation as well as a period of time when the Amendment 2 legislation was approved, but not yet fully implemented.

The data elements collected on the initial treatment plan forms are the date of treatment plan submission and information on the patient, provider, and the cannabis order. All treatment forms were automatically de-identified upon electronic submission. For each patient, a unique registry identification number was generated for longitudinal tracking. Patient data collected were demographics (i.e., age, race/ethnicity), clinical history, medical conditions, history of substance use (i.e., alcohol, tobacco, illicit drugs), prescription medication history, and a patient’s health condition score assessed by the provider on a scale of 1–7 which is based on the Clinical Global Impression Severity Scale [15]. The clinical history included the indication or indications for cannabis treatment, herein referred to as the chief complaint. For the cannabis order, information covered the date of order, the dosing regimen, the type of cannabis (CBD-only, THC + CBD, or both types of products), the planned duration, the treatment plan goal and the plan for monitoring of patient’s symptoms, and a planned follow-up encounter date.

Data collected on the follow-up treatment form include the patient’s registry identification number, the date of treatment plan submission, the date of last patient encounter, changes in the cannabis order since last treatment plan, changes in the chief complaint, hospitalization history since last treatment plan, changes in the patient’s comorbidities or current medications since last treatment plan, indicators of tolerance or reaction to cannabis, discontinuation of cannabis use during the last quarter, and the provider’s assessed patient condition score compared with the initial condition on a scale of 1–7.

We analyzed all electronically submitted forms of registry patients aged 50 to 100 years who attended at least an initial visit. Forms were excluded when providers submitted blank forms and when data entries were erroneous or invalid. Free-text data entries, such as chief complaints, medical treatments, and planned treatment duration were manually reviewed and summarized into clinically meaningful categories. Chief complaint categories were based on the medical conditions listed in the current law and on broader disease categories found to be prominent. Categories for medications were determined by therapeutic classifications that were found to be prominent. The planned treatment duration was categorized into appropriate time intervals determined by all possible entries.

### Data Analysis

We calculated descriptive statistics of patient and treatment characteristics and examined frequency counts, sample means, and proportions. Analyses were conducted with SAS version 9.4 statistical software (SAS Institute Inc, Cary, NC, USA). This study was approved by the institutional review and privacy board of the University of Florida with a waiver of informed consent and HIPAA authorization.

## 3. Results

There were *N* = 4447 older adults registered in Florida’s medical marijuana treatment registry of a total of 7548 registered (Table 1). Of these, 2662 (59.9%) were 50–64, 1238 (27.8%) were 65–74, and 547 (12.3%) were 75 years old or older. Registered users were predominantly of white race (87.5%). Physician-assessed conditions indicated that most patients were moderately ill or worse and low-THC cannabis (i.e., CBD) was the most common treatment choice (45%) compared to medical cannabis (33.3%) or a combination of the two (21.7%). Most patients were given a planned duration of treatment of 12 months or less.

Chief complaints indicating medical marijuana use were primarily related to pain including musculoskeletal disorders, spasms, and chronic pain (Table 2). Cancer was indicated for 15.5% of all patients. Non-pain-related conditions included epilepsy or seizures (2.9%), glaucoma (2%), autoimmune disorders (3.2%) post-traumatic stress disorder (10%), multiple sclerosis (2.7%), Parkinson’s disease (4.5%), amyotrophic lateral sclerosis (ALS; 0.5%) and Crohn’s disease (1.2%). Psychological disorders were prevalent in 13.2% of patients and post-traumatic stress disorder in an additional 10%. These chief complaints were not mutually exclusive, and providers could have identified more than one per patient. Other conditions, such as sleep disorders (7%) and headaches or migraines (10.4%), were also common.

On average, registered patients used approximately 2.5 other medications with medians of 3 (interquartile range 0–4) for all age groups (Table 3). With regards to concomitant medication use, more than 20% of all patients utilized antidepressants (23.8%), anxiolytics and benzodiazepines (23.5%), opioids (28.6%), and cardiovascular agents (27.9%). Among all drug classes with potential sedating effects, 44.8% of the cohort were exposed to at least one.

**Figure A1:**
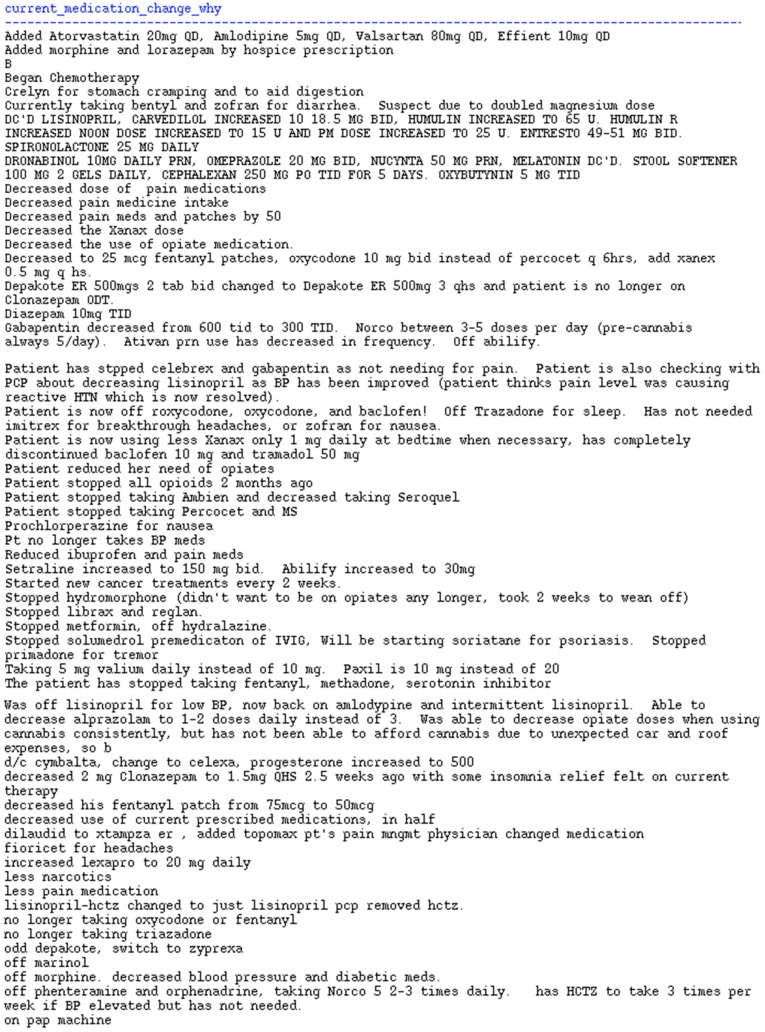
Reasons for Change in Current Medication Noted During Follow-Up Visits; *N* = 85 entries.

**Figure A2:**
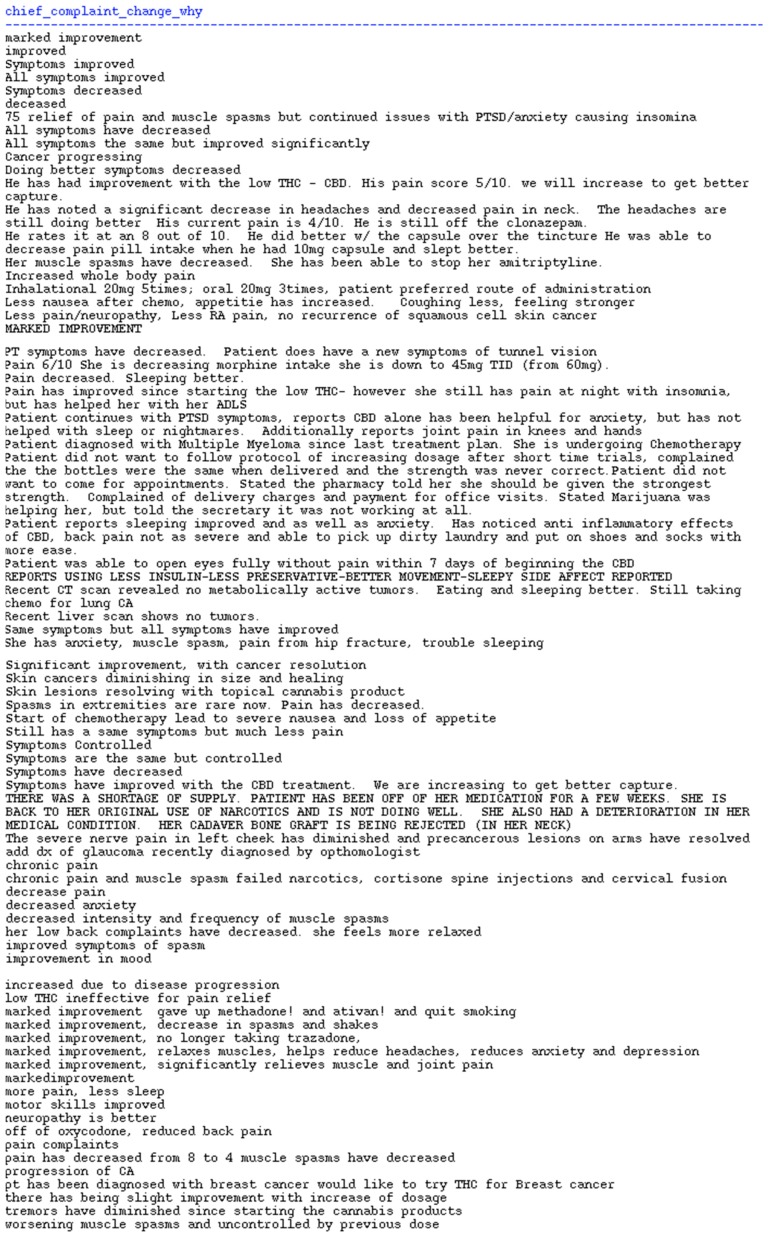
Changes in the Original Chief Complain/Indicated Condition During Follow-Up Visits; *N* = 95 entries.

**Figure A3:**
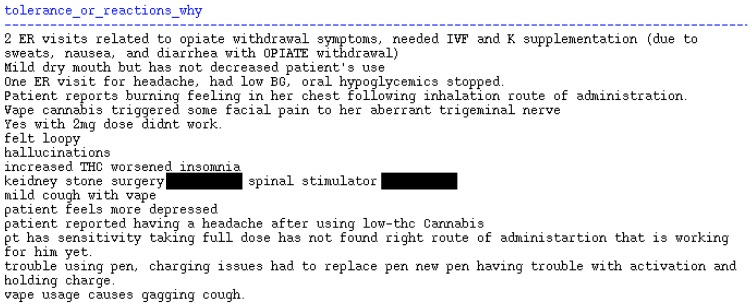
Documentation of Adverse Drug Reactions to Cannabis or Other Patient-Reported Problems with Treatment During Follow-Up Visits; *N* = 16 entries.

**Figure A4:**
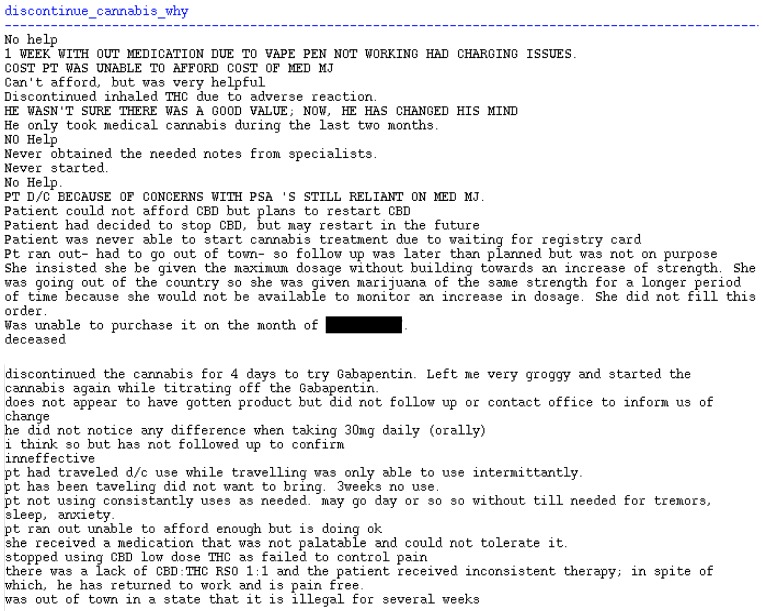
Reasons for Discontinuation of Cannabis During Follow-up Visits; *N* = 33 entries.

Of the 4447 with an initial visit, only 1225 (27.5%) of patients had a second visit treatment plan recorded (Table 4). The majority (72.7%) of patients were recorded to have an improved chief complaint with less than 3% with a worsened complaint. In open response feedback, available for only 85 visits, physicians noted several instances of reduced medication use since initiation of medical marijuana. Noteworthy, were mentions of reduced or stopped opioid medications, improved sleep quality, reduction of medications for sleep, and reduced anxiety medications. Adverse effects were also noted in 16 entries, which included hallucinations, respiratory side effects due to vaped products, sedation or “loopy” feelings, and worsened insomnia. Further, 33 entries were noted in patients who discontinued medical marijuana, which primarily noted inability to afford treatment, preference to not travel with a potentially illegal product, and ineffective treatment. Free text submissions are shown in the Appendix A Figure A1, Figure A2, Figure A3 and Figure A4.

## 4. Discussion

In the state of Florida, there were relatively few initiators of medical marijuana in the first years of implementation but more than one-half were older adults aged ≥50 years. The chief complaints indicating medical marijuana use were primarily related to pain conditions. Other recorded medication use was common and, notably, nearly one-half of registered patients use other potentially sedating medications. Adherence to treatment appeared low, with approximately 1 in 4 patients having a recorded follow-up visit, though the brief treatment plan collection window may not have captured all follow-up visits.

Early adopters may not be completely generalizable to more contemporary late adopters in lifestyle and clinical factors. Nevertheless, several noteworthy concerns are evident even in this sample. Both THC and CBD containing products have high potential to induce side effects of sedation, lethargy, or other altered mental states. In our cohort, nearly one-half used at least one medication such as antidepressants, anti-anxiety, and other classes known to cause sedation. In older adults, this is particularly troubling due to increased sensitivity and higher incidence of negative sequelae that are related to sedation (e.g., falls and fractures). Further, THC-containing pharmaceutical products in other non-U.S. countries are contraindicated in patients with heart disease [16]. In our sample, nearly 1 in 3 patients used cardiovascular medications and may be at risk for additional complications with medical marijuana treatment. Improvements noted in this cohort for the chief complaints as well as reductions in other medications deserve additional research to understand if this is causal. Alternative reasons these improvements were observed may include natural disease progression or attrition of those who did not experience benefit.

The public seems to assume the safety of cannabis and its constituents from a long history of recreational use in mostly younger persons or personal use earlier in life [17,18]. Cannabis is generally viewed as a safer alternative to prescription drugs due to its natural origin and because it has become ubiquitous throughout the U.S. via medical and recreational legalization [19]. Safety is further assumed given that consumer CBD-based products are widely available over the counter for recreational and complementary health uses. However, cannabis has been found to have low-quality evidence for any benefit in a myriad of conditions but has been associated with up to 3-fold higher odds of experiencing adverse drug effects [20,21].

Cannabis is a complex botanical product with broad pharmacologic activity and effects on other medications. Whole cannabis and hemp (with low THC composition) plants contain more than 500 phytoconstituents including, but not limited to, approximately 120 cannabinoids [22,23]. Cannabis-derived substances like CBD are delivered as a purified product, cannabinoid combinations (e.g., CBD:THC) or consumed as part of the whole cannabis or hemp plant [22,23]. Alone, the main cannabinoids CBD and THC have established metabolic routes, absorption/elimination characteristics, and known interactions with drug metabolizing enzymes. Thus, cannabis has potential to cause pharmacokinetic drug–drug interactions as either an inhibitor or inducer of these enzymes [24,25]. Cannabinoids have similar pharmacodynamic properties as many common medications. Constituents in cannabis have significant biological effects, e.g., sedation and somnolence, which can be potentiated with concomitant medications with similar effects (e.g., opioids or benzodiazepines), specifically referred to as pharmacodynamic drug–drug interactions [26,27]. These effects are characterized as both the target effects (e.g., pain relief) that drive patients to seek therapy with cannabis as well as adverse drug events (ADEs) related to cannabis and its components (e.g., psychiatric events). These have included somnolence, sedation, acute psychiatric events (paranoia, hallucination, euphoria), cognitive and memory impairment, insomnia, gait disturbances, suicidal thoughts or behaviors, tachycardia, vertigo, and anorexia [16,21,28,29].

ADEs are a major concern among older adults. Older adults are at an increased risk of ADEs due to pathophysiological changes (e.g., sarcopenia, renal/hepatic dysfunction), polypharmacy, and comorbid conditions [30,31]. The aging brain loses significant volume per decade and places older adults at more susceptibility to neurological ADEs as well as the effects of illicit drugs—including cannabis [32]. Older adults (>50 years) are the largest consumers of prescription medications with 67% using ≥5 prescription drugs, 40% using at least one over-the-counter drug, 60% using a dietary supplement—all numbers which increase throughout aging [33]. In older adults, the estimated prevalence of at least one potential drug–drug interactions in current regimens is 50% and is as high as 80% in certain clinical groups, with up to 1 in 4 patients at risk for ≥4 drug–drug interactions [34,35,36,37]. Many prescription drugs have unclear risk/benefit profiles in older users and have led to clinical tools (e.g., Beers Criteria, STOPP/START, anticholinergic burden scales) [38,39,40,41] to avoid certain medications or avoid specific drug–disease interactions in order to minimize ADEs. In older adults, ADEs disproportionately contribute to severe health outcomes. ADEs are associated with between 3% and 30% of all hospital admissions and ADEs increase the risk of emergency department visits, increased in-hospital morbidity and mortality, and increased health care expenditure [38,41,42,43,44,45,46,47,48,49,50]. It is estimated that up to 50% of all ADEs are avoidable, preventable, or ameliorable in that they can either be prevented through selecting alternative therapies to avoid drug–drug interactions or can be reduced through dose reductions or preventive measures against side effects [44,51,52,53]. The addition of medical cannabis to the armamentarium of treatments for a variety of conditions in older adults deserves further research not only for its potential benefits, but also to fully assess the risks associated with ADEs.

### Limitations

This study included a convenience cohort of older adult medical cannabis users in Florida captured via a physician-provided treatment plan registry. The registry was discontinued due to statutory changes, which did not allow sufficient follow up of patients. Limited patient information was available such as comorbid conditions and medication use, which may have been underreported in the registry. Few follow-up treatment plans were submitted and, thus, assessment of patient outcomes including improvements or adverse effects was not thorough. A new patient registry will be developed in Florida by the Consortium of Medical Marijuana Clinical Outcomes Research, established by state legislature in 2019, to enable better data capture and linkage to other clinical outcome data to improve these limitations.

## 5. Conclusions

Older adults made up more than one-half of all early adopters of medical cannabis. Chronic pain was the most common treatment indication. Registered users were also prescribed several other medications which point to possibilities of drug–drug and drug–disease interactions. Follow up was limited and was likely due to a number of factors including a limited follow-up time, physician non-compliance submitting treatment plans, patients discontinuing medical cannabis, or patient death. Among patients with a follow-up treatment plan, most reported improved conditions and reductions of other medications but some reported side effects or lack of treatment effects. Further research is needed to fill knowledge gaps regarding the safety and effectiveness of medical cannabis for the myriad conditions for which it is being utilized by older patients.

## Figures and Tables

**Table 1:** Characteristics of Florida medical marijuana registry patients at the initial treatment visit by cannabis type ordered.

		Age Group		
Characteristic, *N* (%)	Total (*N* = 4447)	50–64 Years (*N* = 2662)	65–74 Years (*N* = 1238)	75+ Years (*N* = 547)
Age, mean (SD)	63.4 (9.17)	57.3 (4.17)	68.8 (2.71)	80.9 (5.37)
Race				
White	3893 (87.5)	2290 (86.0)	1115 (90.1)	488 (89.2)
Black	157 (3.5)	118 (4.4)	29 (2.3)	***
Hispanic, Latino or Spanish	203 (4.6)	121 (4.6)	52 (4.2)	30 (5.5)
Other/Unknown ^‡^	194 (4.4)	133 (5.0)	42 (3.4)	19 (3.5)
Patient condition assessed by provider				
Normal, not at all ill	195 (4.4)	111 (4.1)	61 (4.9)	23 (4.2)
Borderline ill	99 (2.2)	59 (2.2)	20 (1.6)	20 (3.7)
Mildly ill	588 (13.2)	359 (13.5)	167 (13.5)	62 (11.3)
Moderately ill	1909 (42.9)	1150 (43.2)	512 (41.4)	247 (45.1)
Markedly ill	1156 (26.0)	715 (26.9)	317 (25.6)	124 (22.7)
Severely ill	412 (9.3)	224 (8.4)	130 (10.5)	58 (10.6)
Among the most extremely ill	88 (2.0)	44 (1.7)	31 (2.5)	13 (2.4)
History of substance use				
Alcohol	628 (14.1)	406 (15.3)	160 (12.9)	62 (11.3)
Smoking	444 (10.0)	323 (12.1)	202 (8.2)	20 (3.7)
Illicit drugs	162 (3.6)	118 (4.4)	42 (3.4)	***
Cannabis type ordered ^±^				
Medical cannabis	1481 (33.3)	926 (34.8)	409 (33.1)	146 (26.7)
Low-THC cannabis	2000 (45.0)	1172 (44.0)	534 (43.1)	294 (53.7)
Both low-THC and medical cannabis	966 (21.7)	564 (21.2)	295 (23.8)	107 (19.6)
Planned order duration				
<1 month	469 (10.6)	288 (10.8)	110 (8.9)	71 (13.0)
1–3 months	1919 (43.2)	1209 (45.4)	515 (41.6)	195 (35.7)
3–12 months	382 (8.6)	238 (8.9)	109 (8.8)	35 (6.4)
>12 months or indefinitely	1343 (30.2)	739 (27.8)	406 (32.8)	198 (36.2)
Not specified	334 (7.5)	188 (7.1)	98 (7.9)	48 (8.8)

^‡^ Includes Asian, Native Hawaiian, Pacific Islander, American Indian, or Alaska Native. SD = standard deviation. ^±^ Medical cannabis not explicitly defined by Florida law. Low-THC cannabis defined by Florida law as “containing no more than 0.8 percent of tetrahydrocannabinol (THC) and at least 10 percent of cannabidiol (CBD)”. *** cell count ≤ 10.

**Table 2:** Characteristics of Florida medical marijuana registry patients at the initial treatment visit by cannabis type ordered.

		Age Group		
Chief Complaint ^†^, *N* (%)	Total (*N* = 7548)	50–64 Years (*N* = 2662)	65–74 Years (*N* = 1238)	75+ Years (*N* = 547)
Musculoskeletal disorders and spasms	2154 (48.4)	1348 (50.6)	534 (43.1)	272 (49.7)
Cancer	691 (15.5)	350 (13.2)	235 (19.0)	106 (19.4)
Epilepsy or seizures	130 (2.9)	93 (3.5)	30 (2.4)	***
Glaucoma	87 (2.0)	41 (1.5)	30 (2.4)	16 (2.9)
Autoimmune or immune disorders ^±^	142 (3.2)	104 (3.9)	29 (2.3)	***
Post-traumatic stress disorder (PTSD)	444 (10.0)	298 (11.2)	136 (11.0)	***
Amyotrophic lateral sclerosis (ALS)	24 (0.5)	***	***	***
Crohn’s disease	52 (1.2)	33 (1.2)	15 (1.2)	***
Parkinson’s disease	201 (4.5)	51 (1.9)	92 (7.4)	58 (10.6)
Multiple sclerosis (MS)	121 (2.7)	***	***	***
Chronic pain	2019 (45.4)	1242 (46.7)	520 (42.0)	257 (47.0)
Back, spine, or neck conditions	696 (15.7)	475 (17.8)	147 (11.9)	74 (13.5)
Major brain and head injuries	149 (3.4)	***	***	***
Gastrointestinal conditions	225 (5.1)	137 (5.2)	69 (5.6)	19 (3.5)
Headaches or migraines	461 (10.4)	318 (12.0)	93 (7.5)	50 (9.1)
Other nervous system and neurological disorders	486 (10.9)	269 (10.1)	123 (9.9)	94 (17.2)
Psychological disorders (excl. PTSD)	589 (13.2)	376 (14.1)	158 (12.8)	55 (10.1)
Sleep disorders	310 (7.0)	199 (7.5)	82 (6.6)	29 (5.3)
Others	35 (0.8)	***	***	***

^†^ Chief complaints are not mutually exclusive; more than one condition per patient possible. ^±^ Including HIV/AIDS; excluding MS and Crohn’s disease. *** Data suppressed due to low cell count < 11.

**Table 3:** All concomitant prescription medication classes reported to be used by Florida medical marijuana registry patients at the initial treatment visit ^†^.

		Age Group		
Medication Class, *N* (%)	Total (*N* = 4447)	50–64 Years (*N* = 2662)	65–74 Years (*N* = 1238)	75+ Years (*N* = 547)
Number of medications per patient, mean (SD), IQR	2.4 (2.54) 3 (0–4)	2.4 (2.52) 3 (0–4)	2.4 (2.57) 3 (0–4)	2.3 (2.58) 3 (0–4)
Antidepressants	1060 (23.8)	670 (25.2)	289 (23.4)	101 (18.5)
Antipsychotics	128 (2.9)	82 (3.1)	35 (2.8)	11 (2.0)
Anxiolytics and benzodiazepines	1046 (23.5)	674 (25.3)	285 (23.0)	87 (15.9)
Mood stabilizers	37 (0.8)	***	***	***
Stimulants and amphetamines	124 (2.8)	***	***	***
Hypnotics and sedatives	292 (6.6)	168 (6.3)	98 (7.9)	26 (4.8)
Opioids ^±^	1271 (28.6)	863 (32.4)	296 (23.9)	112 (20.5)
Non-opioid analgesics	861 (19.4)	512 (19.2)	229 (18.5)	120 (21.9)
Skeletal muscle relaxants	611 (13.7)	458 (17.2)	127 (10.3)	26 (4.8)
Other musculoskeletal agents ^††^	133 (3.0)	73 (2.7)	38 (3.1)	22 (4.0)
Anticonvulsants and antiepileptics	760 (17.1)	496 (18.6)	176 (14.2)	88 (16.1)
Anti-Parkinson	162 (3.6)	58 (2.2)	69 (5.6)	35 (6.4)
Other neurological agents ^±±^	71 (1.6)	39 (1.5)	21 (1.7)	11 (2.0)
Antiemetics	200 (4.5)	128 (4.8)	56 (4.5)	16 (2.9)
Other GI agents	217 (4.9)	135 (5.1)	58 (4.7)	24 (4.4)
Cardiovascular agents	1241 (27.9)	623 (23.4)	417 (33.7)	201 (36.8)
Antidiabetic agents	271 (6.1)	147 (5.5)	92 (7.4)	32 (5.9)
Hematologic agents	126 (2.8)	52 (2.0)	51 (4.1)	23 (4.2)
Hormonal agents and steroids	596 (13.4)	319 (12.0)	198 (16.0)	79 (14.4)
Genitourinary agents	264 (5.9)	99 (3.7)	100 (8.1)	65 (11.9)
Respiratory agents	181 (4.1)	90 (3.4)	60 (4.9)	31 (5.7)
Chemotherapeutic agents	102 (2.3)	***	***	***
Autoimmune agents	75 (1.7)	***	***	***
Antivirals incl. HIV medications	40 (0.9)	***	***	***
Anti-infective agents	50 (1.1)	***	***	***
Ophthalmic and glaucoma medications	51 (1.2)	17 (0.6)	18 (1.5)	16 (2.9)
OTC medications, vitamins, supplements and others	348 (8.2)	204 (7.6)	111 (9.0)	48 (8.8)

SD = standard deviation; IQR = interquartile range. ^†^ Medications are not mutually exclusive, more than one medication per patient possible. ^±^ Includes combination products containing an opioid; ^††^ Includes medications for multiple sclerosis. ^±±^ Includes triptans and medications for Alzheimer’s disease. *** Data suppressed due to low cell count < 11.

**Table 4:** Summary of the follow-up information reported by Florida medical marijuana registry patients at a follow-up visit after treatment initiation for total follow-up sample.

Total (*N* = 1225)	Yes (%)
**Follow-Up Question Since Last Treatment Visit**	
Changes in chief complaint since last visit?	10.0%
Changes in alcohol, smoking, or illicit drug use since last visit? ^†^	1.4%
Changes in comorbidities since last visit?	1.7%
Hospitalizations since last visit?	2.9%
Changes in current medications since last visit?	10.0%
Were there indicators of reaction to cannabis since last visit? ^‡^	2.0%
Did the patient discontinue cannabis use?	4.6%
**Patient Condition Since the Initiation of Treatment Compared to Condition Initially Assessed**	
Very much improved	10.8%
Much improved	31.4%
Minimally improved	30.5%
No change from baseline	24.7%
Minimally worse	1.4%
Much worse	0.9%
Very much worse	0.4%

^†^ Missing *N* = 62. ^‡^ Adverse drug reactions, patient-reported problems, medications holds, ER visits, or hospitalizations.

## Appendix A

Free-text entries of follow-up treatment plans indicating changes in chief complaints, medications, and patient experiences after an initial treatment with medical marijuana (Figure A1, Figure A2, Figure A3 and Figure A4).

## References

[1] National Conference of State Legislatures (NCSL) State Medical Marijuana Laws. http://www.ncsl.org/research/health/state-medical-marijuana-laws.aspx.

[2] Denham B.E. (2019). Attitudes toward legalization of marijuana in the United States, 1986–2016: Changes in determinants of public opinion. Int. J. Drug Policy.

[3] Cerda M., Wall M., Keyes K.M., Galea S., Hasin D. (2012). Medical marijuana laws in 50 states: Investigating the relationship between state legalization of medical marijuana and marijuana use, abuse and dependence. Drug Alcohol Depend..

[4] Number of Legal Medical Marijuana Patients. https://medicalmarijuana.procon.org/view.resource.php?resourceID=005889.

[5] Compton W.M., Han B., Hughes A., Jones C.M., Blanco C. (2017). Use of marijuana for medical purposes among adults in the United States. JAMA.

[6] Wong S.S., Wilens T.E. (2017). Medical Cannabinoids in children and adolescents: A systematic review. Pediatrics.

[7] Sexton M., Cuttler C., Finnell J.S., Mischley L.K. (2016). A Cross-sectional survey of medical cannabis users: Patterns of use and perceived efficacy. Cannabis Cannabinoid Res..

[8] Lucas P., Walsh Z. (2017). Medical cannabis access, use, and substitution for prescription opioids and other substances: A survey of authorized medical cannabis patients. Int. J. Drug Policy.

[9] Han B.H., Sherman S., Mauro P.M., Martins S.S., Rotenberg J., Palamar J.J. (2017). Demographic trends among older cannabis users in the United States, 2006–2013. Addiction.

[10] Lin L.A., Ilgen M.A., Jannausch M., Bohnert K.M. (2016). Comparing adults who use cannabis medically with those who use recreationally: Results from a national sample. Addict. Behav..

[11] Dai H., Richter K.P. (2019). A national survey of marijuana use among us adults with medical conditions, 2016–2017. JAMA Netw. Open.

[12] Ellis J.D., Resko S.M., Szechy K., Smith R., Early T.J. (2019). Characteristics associated with attitudes toward marijuana legalization in Michigan. J. Psychoact. Drugs.

[13] Briscoe J., Casarett D. (2018). Medical marijuana use in older adults. J. Am. Geriatr. Soc..

[14] Lloyd S.L., Striley C.W. (2018). Marijuana use among adults 50 years or older in the 21st century. Gerontol. Geriatr. Med..

[15] Guy W. Clinical global impression (CGI). https://www.psywellness.com.sg/docs/CGI.pdf.

[16] Sativex(R) (2005). Delta-9-Tetrahydrocannabinol and Cannabidiol.

[17] Hall W., Renstrom M., Poznyak V. (2016). The Health and Social Effects of Nonmedical Cannabis Use.

[18] Hall W., Weier M. (2015). Assessing the public health impacts of legalizing recreational cannabis use in the USA. Clin. Pharm..

[19] Resko S., Ellis J., Early T.J., Szechy K.A., Rodriguez B., Agius E. (2019). Understanding public attitudes toward cannabis legalization: Qualitative findings from a statewide survey. Subst. Use Misuse.

[20] Volkow N.D., Baler R.D., Compton W.M., Weiss S.R. (2014). Adverse health effects of marijuana use. N. Engl. J. Med..

[21] Whiting P.F., Wolff R.F., Deshpande S., Di Nisio M., Duffy S., Hernandez A.V., Keurentjes J.C., Lang S., Misso K., Ryder S. (2015). Cannabinoids for medical use: A systematic review and meta-analysis. JAMA.

[22] ElSohly M.A., Radwan M.M., Gul W., Chandra S., Galal A. (2017). Phytochemistry of cannabis sativa L.. Prog. Chem. Org. Nat. Prod..

[23] Elsohly M.A., Slade D. (2005). Chemical constituents of marijuana: The complex mixture of natural cannabinoids. Life Sci..

[24] Jiang R., Yamaori S., Takeda S., Yamamoto I., Watanabe K. (2011). Identification of cytochrome P450 enzymes responsible for metabolism of cannabidiol by human liver microsomes. Life Sci..

[25] Brown J.D., Winterstein A.G. (2019). Potential adverse drug events and drug-drug interactions with medical and consumer cannabidiol (CBD) use. J. Clin. Med..

[26] Bergamaschi M.M., Queiroz R.H., Zuardi A.W., Crippa J.A. (2011). Safety and side effects of cannabidiol, a cannabis sativa constituent. Curr. Drug. Saf..

[27] Iffland K., Grotenhermen F. (2017). An update on safety and side effects of cannabidiol: A review of clinical data and relevant animal studies. Cannabis Cannabinoid Res..

[28] GW Research Ltd. Drug Approval Package: Epidiolex (Cannabidiol). https://www.accessdata.fda.gov/drugsatfda_docs/nda/2018/210365Orig1s000TOC.cfm.

[29] Brown J.D. (2020). Potential adverse drug events with tetrahydrocannabinol (THC) due to drug–drug interactions. J. Clin. Med..

[30] Corsonello A., Pedone C., Incalzi R.A. (2010). Age-related pharmacokinetic and pharmacodynamic changes and related risk of adverse drug reactions. Curr. Med. Chem..

[31] Trifiro G., Spina E. (2011). Age-related changes in pharmacodynamics: Focus on drugs acting on central nervous and cardiovascular systems. Curr. Drug Metab..

[32] Dowling G.J., Weiss S.R., Condon T.P. (2008). Drugs of abuse and the aging brain. Neuropsychopharmacology.

[33] Qato D.M., Alexander G.C., Conti R.M., Johnson M., Schumm P., Lindau S.T. (2008). Use of prescription and over-the-counter medications and dietary supplements among older adults in the united states. JAMA.

[34] Schneider K.L., Kastenmuller K., Weckbecker K., Bleckwenn M., Bohme M., Stingl J.C. (2018). Potential drug-drug interactions in a cohort of elderly, polymedicated primary care patients on antithrombotic treatment. Drugs Aging.

[35] Tulner L.R., Frankfort S.V., Gijsen G.J., van Campen J.P., Koks C.H., Beijnen J.H. (2008). Drug-drug interactions in a geriatric outpatient cohort: Prevalence and relevance. Drugs Aging.

[36] Nobili A., Pasina L., Tettamanti M., Lucca U., Riva E., Marzona I., Monesi L., Cucchiani R., Bortolotti A., Fortino I. (2009). Potentially severe drug interactions in elderly outpatients: Results of an observational study of an administrative prescription database. J. Clin. Pharm..

[37] Johnell K., Klarin I. (2007). The relationship between number of drugs and potential drug-drug interactions in the elderly: A study of over 600,000 elderly patients from the Swedish prescribed drug register. Drug Saf..

[38] Brown J.D., Hutchison L.C., Li C., Painter J.T., Martin B.C. (2016). Predictive validity of the beers and screening tool of older persons’ potentially inappropriate prescriptions (STOPP) criteria to detect adverse drug events, hospitalizations, and emergency department visits in the United States. J. Am. Geriatr. Soc..

[39] (2019). American geriatrics society 2019 updated AGS beers criteria(R) for potentially inappropriate medication use in older adults. J. Am. Geriatr. Soc..

[40] O’Mahony D., O’Sullivan D., Byrne S., O’Connor M.N., Ryan C., Gallagher P. (2015). STOPP/START criteria for potentially inappropriate prescribing in older people: Version 2. Age Ageing.

[41] Rudolph J.L., Salow M.J., Angelini M.C., McGlinchey R.E. (2008). The anticholinergic risk scale and anticholinergic adverse effects in older persons. Arch. Intern. Med..

[42] Budnitz D.S., Pollock D.A., Weidenbach K.N., Mendelsohn A.B., Schroeder T.J., Annest J.L. (2006). National surveillance of emergency department visits for outpatient adverse drug events. JAMA.

[43] Budnitz D.S., Shehab N., Kegler S.R., Richards C.L. (2007). Medication use leading to emergency department visits for adverse drug events in older adults. Ann. Intern. Med..

[44] Gurwitz J.H., Field T.S., Harrold L.R., Rothschild J., Debellis K., Seger A.C., Cadoret C., Fish L.S., Garber L., Kelleher M. (2003). Incidence and preventability of adverse drug events among older persons in the ambulatory setting. JAMA.

[45] Budnitz D.S., Lovegrove M.C., Shehab N., Richards C.L. (2011). Emergency hospitalizations for adverse drug events in older Americans. N. Engl. J. Med..

[46] Brown J.D., Painter J., Li C., Hutchison L.C., Martin B. (2014). Adverse drug events in the elderly occurring in emergency, inpatient, and outpatient departments in an administrative claims database. Value Health.

[47] Hanlon J.T., Schmader K.E., Koronkowski M.J., Weinberger M., Landsman P.B., Samsa G.P., Lewis I.K. (1997). Adverse drug events in high risk older outpatients. J. Am. Geriatr. Soc..

[48] Poudel D.R., Acharya P., Ghimire S., Dhital R., Bharati R. (2017). Burden of hospitalizations related to adverse drug events in the USA: A retrospective analysis from large inpatient database. Pharm. Drug Saf..

[49] Riaz M., Brown J.D. (2019). Association of adverse drug events with hospitalization outcomes and costs in older adults in the USA using the nationwide readmissions database. Pharm. Med..

[50] Winterstein A.G., Sauer B.C., Hepler C.D., Poole C. (2002). Preventable drug-related hospital admissions. Ann. Pharm..

[51] Von Laue N.C., Schwappach D.L., Koeck C.M. (2003). The epidemiology of preventable adverse drug events: A review of the literature. Wien. Klin. Wochenschr..

[52] Thomsen L.A., Winterstein A.G., Sondergaard B., Haugbolle L.S., Melander A. (2007). Systematic review of the incidence and characteristics of preventable adverse drug events in ambulatory care. Ann. Pharm..

[53] Kanjanarat P., Winterstein A.G., Johns T.E., Hatton R.C., Gonzalez-Rothi R., Segal R. (2003). Nature of preventable adverse drug events in hospitals: A literature review. Am. J. Health Syst. Pharm..

